# Apathy is associated with large-scale white matter network disruption in small vessel disease

**DOI:** 10.1212/WNL.0000000000007095

**Published:** 2019-03-12

**Authors:** Jonathan Tay, Anil M. Tuladhar, Matthew J. Hollocks, Rebecca L. Brookes, Daniel J. Tozer, Thomas R. Barrick, Masud Husain, Frank-Erik de Leeuw, Hugh S. Markus

**Affiliations:** From the Department of Clinical Neurosciences (J.T., M.J.H., R.L.B., D.J.T., H.S.M.), University of Cambridge, UK; Department of Neurology (A.M.T., F.-E.d.L.), Donders Institute for Brain, Cognition and Behaviour, Radboud University Nijmegen Medical Centre, Nijmegen, the Netherlands; Neuroscience Research Centre (T.R.B.), Molecular and Clinical Sciences Research Institute, St. George's University of London; and Nuffield Department of Clinical Neurosciences (M.H.), University of Oxford, UK.

## Abstract

**Objective:**

To investigate whether white matter network disruption underlies the pathogenesis of apathy, but not depression, in cerebral small vessel disease (SVD).

**Methods:**

Three hundred thirty-one patients with SVD from the Radboud University Nijmegen Diffusion Tensor and Magnetic Resonance Cohort (RUN DMC) study completed measures of apathy and depression and underwent structural MRI. Streamlines reflecting underlying white matter fibers were reconstructed with diffusion tensor tractography. First, path analysis was used to determine whether network measures mediated associations between apathy and radiologic markers of SVD. Next, we examined differences in whole-brain network measures between participants with only apathy, only depression, and comorbid apathy and depression and a control group free of neuropsychiatric symptoms. Finally, we examined regional network differences associated with apathy.

**Results:**

Path analysis demonstrated that network disruption mediated the relationship between apathy and SVD markers. Patients with apathy, compared to all other groups, were impaired on whole-brain measures of network density and efficiency. Regional network analyses in both the apathy subgroup and the entire sample revealed that apathy was associated with impaired connectivity in premotor and cingulate regions.

**Conclusions:**

Our results suggest that apathy, but not depression, is associated with white matter tract disconnection in SVD. The subnetworks delineated suggest that apathy may be driven by damage to white matter networks underlying action initiation and effort-based decision making.

Apathy is a reduction in goal-directed behavior that manifests as decreased initiative and interest.^1^ It is associated with quality-of-life deficits^2^ and a doubled dementia risk^3^ independently of depression, a negative emotional state that is dissociable from apathy.^4^ Apathy is prevalent in cerebral small vessel disease (SVD),^5^ a vascular pathology that damages white matter and leads to stroke, cognitive decline, and disability.^6^

Understanding the neural basis of apathy may lead to novel approaches for diagnosis and treatment. Neuroanatomic models of apathy suggest that it is the product of lesions to the basal ganglia and prefrontal cortex, leading to disrupted goal-directed behaviour.^7^ However, recent MRI studies have shown that apathy is associated with spatially extensive reductions in white matter microstructural integrity.^2,8,9^ This suggests that apathy may be a disconnection syndrome, although this hypothesis remains untested. Furthermore, related issues, including how SVD pathology leads to apathy and how white matter connectivity differs in patients with apathy and depression, remain largely unknown.

We investigated these questions by evaluating the hypothesis that apathy is a disconnection syndrome. We used diffusion tensor tractography to reconstruct white matter pathways in patients with SVD, which were then analyzed with the use of network analysis. First, we investigated whether lacunar infarcts (LIs) and white matter hyperintensities (WMH) are related to apathy through network disruption. Next, we compared whole-brain networks of patients with apathy to those with depression to characterize the nature of network impairment in apathetic patients. Finally, we localized region-specific network disruption associated with apathy.

## Methods

### Sample

Participants were patients with SVD recruited to the Radboud University Nijmegen Diffusion Tensor and Magnetic Resonance Cohort (RUN DMC) study. RUN DMC is a prospective cohort study with enrolled participants meeting the following inclusion criteria: (1) WMH and/or LI of presumed vascular origin on MRI^10^; (2) age between 50 and 85 years; (3) free of dementia, assessed with DSM-IV-TR criteria,^11^ on recruitment; and (4) no (psychiatric) disease interfering with cognitive testing or follow-up, which included patients with bipolar disorder or schizophrenia. The full protocol has been provided elsewhere.^12^

Baseline data collection occurred in 2006, during which 503 participants were enrolled, 98% of whom identified as white. Follow-up occurred in 2011. The apathy scale we used was not included at baseline, so the data analyzed were from the 2011 follow-up only. Of the 503 participants enrolled at baseline, 105 were lost to follow-up, deceased, or unable to perform an in-person assessment, while 67 were excluded due to issues with the acquisition, quality, or processing of the MRI data, bringing the final sample in our study to 331. Demographic and clinical measures for the study population are reported in table 1.

**Table 1:**
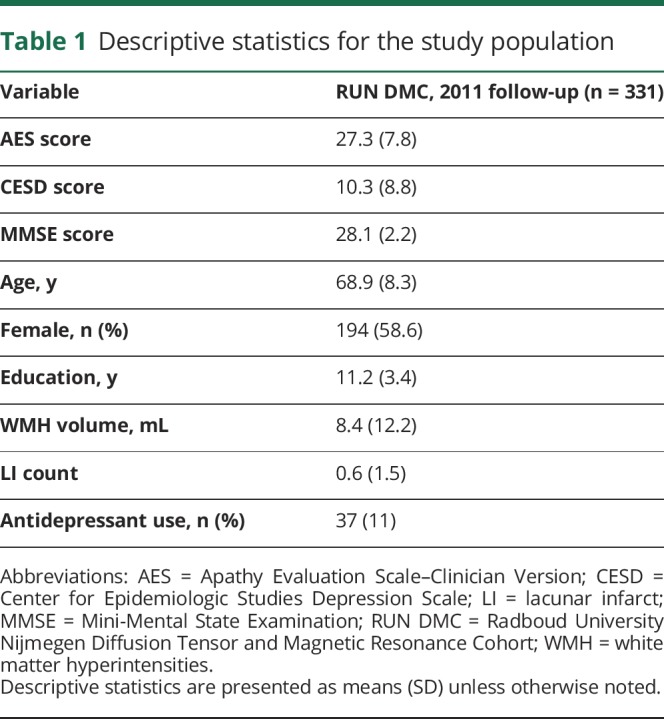
Descriptive statistics for the study population

### Standard protocol approvals, registrations, and patient consents

The study was approved by the Medical Review Ethics Committee Arnhem-Nijmegen, and all participants provided written informed consent.

### Measures

Apathy was measured with the clinician-rated Apathy Evaluation Scale (AES),^13^ an 18-item measure of apathy validated in stroke. Depressive symptoms were measured with the Centre for Epidemiological Studies Depression Scale (CESD),^14^ a reliable and valid 20-item screening instrument for depression^15^ that has been used in other studies of SVD.^16^ For both measures, higher scores indicate higher levels of apathy and depression, respectively. Aside from their psychometric properties, the AES and CESD were chosen for their brevity, which helped minimize patient and administrator burden, a concern given our large sample size and detailed in-person assessment. Cognitive function was evaluated with the Mini-Mental State Examination.^17^ Missing data were imputed with the chained equations technique and predictive mean matching.^18^

Participants were divided into 4 groups based on established cut scores on the AES and CESD.^14,19^ The apathy group was defined as total AES score ≥34 and total CESD score <16. The depression group was defined as AES score <34 and CESD score ≥16. The comorbid apathy and depression group was defined as AES score ≥34 and CESD score ≥16. Remaining participants were assigned to a control group free of apathy and depression.

### MRI acquisition parameters

Images were acquired on a Siemens Magnetom Avanto Tim 1.5T MRI scanner (Erlangen, Germany). The protocol included a T1-weighted 3-dimensional magnetization-prepared rapid gradient-echo image (repetition time [TR] 2,250 milliseconds, echo time [TE] 2.95 milliseconds, inversion time [TI] 850 milliseconds, flip angle 15°, voxel size 1.0 mm isotropic), a fluid-attenuated inversion recovery (FLAIR) sequence (TR 14,240 milliseconds, TE 89 milliseconds, TI 2,200 milliseconds, voxel size 1.2 × 1.0 × 2.5 mm, interslice gap 0.5 mm), and a diffusion-weighted echo-planar imaging (EPI) sequence (TR 10,200 milliseconds, TE 95 milliseconds, voxel size 2.5 mm isotropic; 7 scans with *b* = 0 s/mm^2^, 61 scans with *b* = 900 s/mm^2^).

### Radiologic markers of SVD

WMH volumes were segmented on the FLAIR images with a semiautomatic method.^20^ These were then normalized to each participant's intracranial volume (in milliliters), which was calculated by summing the intensities of the voxels covered by the gray matter, white matter, and CSF tissue probability maps generated through unified segmentation in SPM12 (fil.ion.ucl.ac.uk/spm/). All segmentations were visually inspected for errors. LIs were manually counted on the T1-weighted and FLAIR images by 2 trained raters following the Standards for Reporting Vascular Changes on Neuroimaging criteria.^10^ Both raters were blind to the clinical data, and interrater reliability was excellent (Cohen κ = 0.95).^21^

### Diffusion tensor imaging preprocessing

Raw diffusion data were denoised with a local principal component analysis filter^22^ and then corrected for head movement, cardiac motion, and eddy currents with the PATCH algorithm.^23^ The diffusion-weighted images were realigned to the unweighted diffusion image (the *b*0 image) with mutual information based coregistration in SPM12. EPI distortions were unwarped by normalizing EPI images to the T1-weighted images in the phase-encoding direction with SPM12. FSL (fsl.fmrib.ox.ac.uk/fsl/) was then used to extract brain tissue and to calculate the diffusion tensor. In-house software was used to perform whole-brain deterministic diffusion tensor tractography in each participant's native diffusion space.^24^ Streamlines were seeded at every point in an evenly spaced 0.5-mm^3^ grid and propagated in the orthograde and retrograde directions by interpolating the diffusion tensor field in the principal diffusion direction. Streamlines were terminated when the angle between principal eigenvectors θ was ≥45° or fractional anisotropy was <0.2.^25^

### Network construction

A mathematical network is a series of nodes that are connected by edges. In research on macroscopic anatomic brain networks, gray matter regions are typically used as nodes, while edges are defined as the white matter tracts that connect them.^26^ We adopted these conventions for the construction of our white matter networks.

Network nodes were defined with the Automated Anatomical Labeling (AAL) atlas,^27^ which has been used in other network-based studies in SVD.^25,28,29^ The AAL is a volumetric segmentation of the gray matter from which we extracted 90 regions for our analysis (45 per hemisphere), excluding cerebellar regions. The registration of the AAL atlas to each participant's *b*0 image was carried out with default parameters in Advanced Normalization Tools (stnava.github.io/ANTs/). A linear affine transformation was used to register each participant's *b*0 image to the T1-weighted image, while a symmetric diffeomorphic nonlinear transformation was used to register the T1-weighted images to Montreal Neurological Institute space. The resulting transformation matrices were then inverted, concatenated, and applied to the AAL image, bringing the atlas into each participant's *b*0 space. All registrations were visually inspected for errors. The 90 regions of the AAL atlas were used as network nodes, forming the basis of participant-specific structural connectivity matrices.

Network edges were defined on the basis of connected node pairs. Two nodes, *i* and *j*, were connected by an edge, *e*_ij_, if the endpoints of a tractography-reconstructed streamline lay within both regions. Edges were weighted, *w*(*e*_ij_), by streamline length in millimeters, *l*, such that 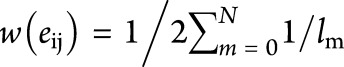, where *N* is the set of streamlines connecting nodes *i* and *j*. This equation scales edge weights to correct for the number of seeds per millimeter because tractography can be seeded at multiple points along the same streamline, yielding inflated edge weights for long-distance connections. Edges were then thresholded at *w*(*e*_ij_) = 1 to eliminate noise-related false-positives. This edge-weighting procedure and thresholding has been used in other tractography-based network analysis studies of SVD.^25,29^ This produced a weighted undirected 90 × 90 connectivity matrix for each participant. Each element of the connectivity matrix therefore represented the corrected number of tractography-reconstructed streamlines connecting 2 gray matter regions delineated by the AAL.

### Graph theoretical analysis

We analyzed 2 core characteristics of whole-brain network connectivity: density and organization. The density of a network is the ratio of observed edges to all possible edges in a network, with the resulting value being a measure of how sparsely connected a network is. To measure the organization of connections, we computed global and local efficiencies. Global efficiency, the average inverse shortest path length in the network, is a measure of connectivity between distal brain structures. The average local efficiency is the global efficiency computed on first-degree neighbors of a node and is a measure of how well connected the local clusters of brain structures are. Efficiency metrics are thought to reflect the ease of communication within a network and can be meaningfully computed on pairs of disconnected nodes,^30^ which are common in SVD.^25^ For both measures, smaller values indicate less efficient brain networks. All whole-brain network measures were computed with the Brain Connectivity Toolbox (brain-connectivity-toolbox.net).

### Statistical analysis

Statistics were calculated with R 3.4 (r-project.org, R Foundation for Statistical Computing, Vienna, Austria). All tests were 2 tailed, thresholded at *p* < 0.05, and corrected for multiple comparisons with the Bonferroni-Holm method. WMH and LI were log transformed to reduce skew.

### Mediation analysis

Bivariate correlations were used to assess the relationships between apathy and all other variables of interest in the study. We then used path analysis to model the mediating effect of network integrity, as measured by global efficiency, on the relationship between SVD markers and apathy. We also tested an alternative mediation model in which SVD markers controlled for the relationship between global efficiency and apathy.

To determine whether network integrity was related to apathy after controlling for other variables, we conducted a multiple linear regression analysis. In this model, apathy was the dependent variable, which was predicted by variables that were associated with it in our bivariate correlation analysis. Variables that remained predictors after this procedure were then carried forward as covariates for our whole-brain and regional network analyses.

### Whole-brain network analysis

One-way analysis of covariance (ANCOVA) was used to test for differences in network density, global efficiency, and local efficiency among the apathy, depression, comorbid, and control groups. Control variables were identified through our earlier multiple regression analysis. Because of unbalanced group sizes, ANCOVAs were computed with type II sums of squares. After a significant result, between-group post hoc comparisons were conducted with the Tukey honest significant difference or the Fisher test.

### Regional network analysis

In contrast to our whole-brain analysis, which investigated how network measures were disrupted, our regional analysis investigated where disruption had occurred, necessitating hypothesis testing at each edge within the connectivity matrices. Because edges are the component tested, inferences are fundamentally pairwise because 1 edge connects a pair of nodes. We conducted 3 such analyses.

For the first analysis, we compared network matrices between the apathy group and the rest of the sample. Significant edges, in this context, reflect connections that differ between the apathy group and the rest of the sample. In other words, this analysis examined the unique network topology, or subnetwork, that characterized patients with apathy.

To leverage our continuous scale data and large sample size, our second and third analyses used all participants, regardless of group membership. For the second analysis, a general linear model was fitted at each edge with AES and CESD scores used as independent variables. Separate contrast vectors were specified to assess the significance of the resulting regression coefficients. The apathy contrast tested for a relationship between apathy and an edge while controlling for depression as a covariate and vice versa. Significant edges, in this context, reflect subnetworks that are associated with apathy after controlling for depression. Our third analysis was similar to the second but included the other covariates identified in our earlier multiple regression analysis.

All regional network analyses were conducted with the Network Based Statistic (NBS) toolbox (nitrc.org/projects/nbs). Edges were deemed significant at t ≥ 3.1 (corresponding approximately to *p* = 0.001), and component sizes were determined from cluster extent. Multiple comparisons were controlled with the NBS, and data were permuted 10,000 times to generate corrected *p* values. Because tests in the NBS are 1 sided, significance levels were adjusted to *p* < 0.025 to test both tails of the distribution.

### Data availability

Anonymized data can be made available to qualified investigators on request to the corresponding author.

## Results

All reported *p* values have been corrected for multiple comparisons as detailed in the Methods.

### Mediation analysis

**Figure 1:**
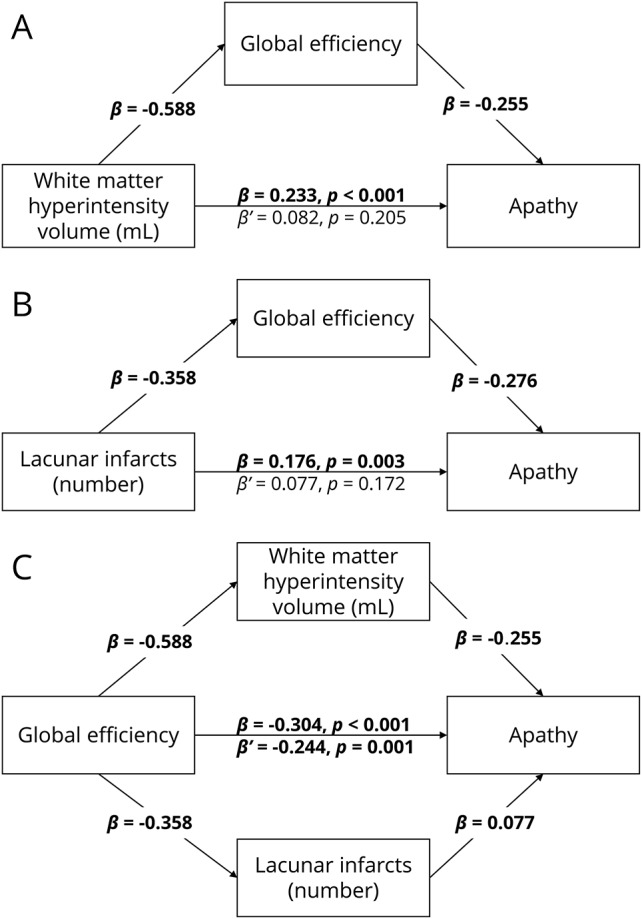
Results of the mediation analyses (A) Mediating effect of global efficiency on the relationship on white matter hyperintensities (WMH) volume and apathy. (B) Mediating effect of global efficiency on the relationship between lacunar infarct (LI) number and apathy. (C) Mediating effect of WMH volume and LI number on the relationship between global efficiency and apathy. All numbers represent standardized β coefficients. β Is the coefficient before mediation; β′ is the coefficient after mediation. Significant paths at *p* < 0.01 after Bonferroni-Holm adjustment are highlighted in bold.

Apathy was correlated with depression, cognition, education, WMH, LIs, and network measures (*p* ≤ 0.01) (table 2). Because all network measures were highly correlated (*r* > 0.85), we used only global efficiency in our mediation analyses.^31^ Path analysis models revealed that the relationship between WMH volume and apathy (β = 0.233, *p* < 0.001) did not remain after controlling for global efficiency (β′ = 0.082, *p* = 0.205) (figure 1A). Similarly, LI number was related to apathy (β = 0.176, *p* = 0.003) but not after controlling for global efficiency (β′ = 0.077, *p* = 0.172) (figure 1B). In contrast, the relationship between global efficiency and apathy, (β = −0.304, *p* < 0.001) remained after controlling for both WMH volume and LIs simultaneously (β′ = −0.244, *p* = 0.001) (figure 1C).

**Table 2:**
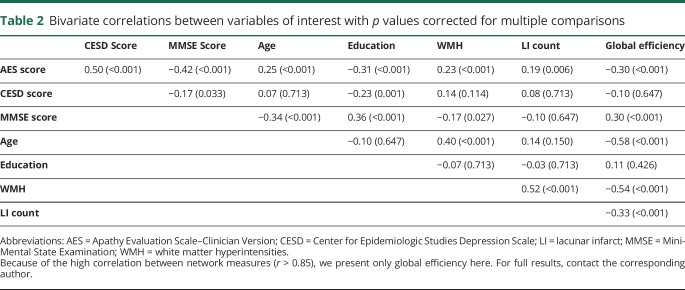
Bivariate correlations between variables of interest with *p* values corrected for multiple comparisons

The multiple regression analysis using all demographic and clinical variables correlated with apathy (table 2) revealed that global efficiency remained a predictor of apathy (β = −0.131, *p* = 0.028), as did depression (β = 0.416, *p* < 0.001), cognition (β = −0.259, *p* < 0.001), and education (β = −0.106, *p* = 0.024). Age, WMH, and LIs were no longer correlated (*p* > 0.05) and were thus removed from further analyses.

### Whole-brain network analysis

Using established cut scores on the AES and CESD, investigators assigned participants to apathy (n = 26), depression (n = 48), comorbid apathy and depression (n = 32), and control SVD (n = 225) groups (table 3). Between-group differences were found for antidepressant use (χ^2^ = 27.187, *p* < 0.001) but not sex (χ^2^ = 4.123, *p* = 0.249). Antidepressant use differed between the depression and control groups (odds ratio 0.238, *p* = 0.006) and comorbid and control groups (odds ratio 0.119, *p* < 0.001). The apathy group was older than both the depression and control groups (*p* < 0.001). The apathy and comorbid groups had lower Mini-Mental State Examination scores compared to the depression and control groups (*p* ≤ 0.01). The apathy and comorbid groups were less educated than the control group (*p* < 0.05).

**Table 3:**
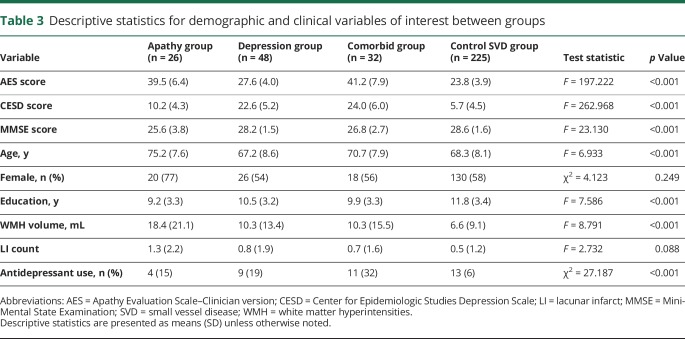
Descriptive statistics for demographic and clinical variables of interest between groups

ANCOVAs comparing whole-brain network measures revealed differences in density, global, and local efficiency (table 4). Post hoc tests revealed that the apathy group scored lower on all network measures compared to the control group, depression group, and comorbid group (table 5). The depression, comorbid, and control groups did not differ on any network measure.

**Table 4:**
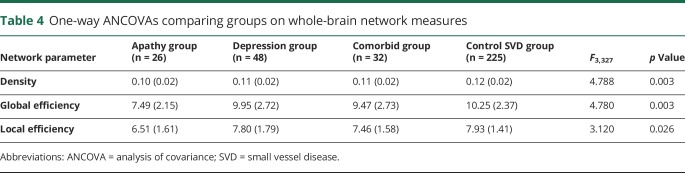
One-way ANCOVAs comparing groups on whole-brain network measures

**Table 5:**
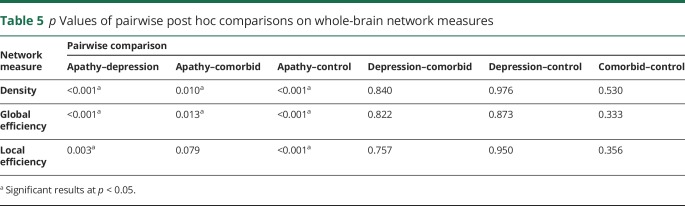
*p* Values of pairwise post hoc comparisons on whole-brain network measures

### Regional network analysis

**Figure 2:**
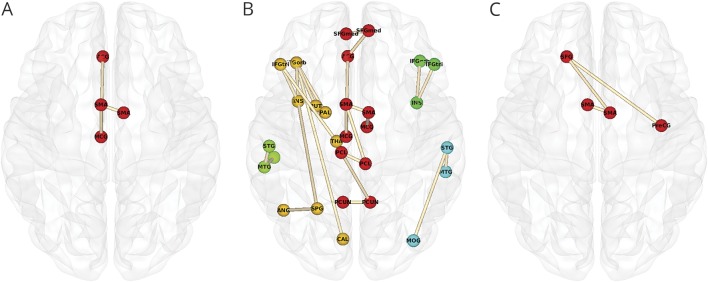
Topologic clusters related to apathy (A) Cluster that differed between the apathy group and all other participants. (B) Clusters associated with apathy in all participants while controlling for depression. Nodes are colored according to the unique clusters they form. See table 5 for a full list of significant edges, grouped by topologic cluster. (C) Clusters associated with apathy in all participants while controlling for depression, cognition, and education. Networks were projected on the Montreal Neurological Institute 152 standard space template and visualized from the axial plane in neurologic convention. CAL = pericalcarine cortex; IFGorb = inferior frontal gyrus pars orbitalis; IFGtri = inferior frontal gyrus pars triangularis; INS = insula; MCG = middle cingulate gyrus; MOG = middle occipital gyrus; MTG = middle temporal gyrus; PAL = palladium; PCL = paracentral lobule; PCUN = precuneus; PreCG = precentral gyrus; PUT = putamen; SFG = superior frontal gyrus; SFGmed = medial superior frontal gyrus; SMA = supplementary motor area; SPG = superior parietal gyrus; STG = superior temporal gyrus; THA = thalamus.

To investigate whether there were regional differences in white matter networks associated with apathy, we first performed an edgewise comparison between the apathy group and the rest of the sample. This revealed a single topologic cluster, with edges connecting the bilateral supplementary motor area (SMA), *t* = 3.932; left SMA to left middle cingulate gyrus (MCG), *t* = 3.277; and left MCG to left anterior cingulate gyrus, *t* = 3.161 (figure 2A).

After our comparison of the apathy only group to the rest of the sample, we conducted an additional pairwise comparison between the apathy only and comorbid groups. This would allow us to determine whether the apathy only group had specific subnetworks that were impaired relative to the comorbid group. Both groups were compared by use of the same criteria in the methods. No between-group regional differences were found.

We then examined edgewise correlations with apathy while controlling for depression and vice versa. Apathy was associated with 5 distinct topologic clusters (table 6 and figure 2B). The first and largest cluster (red in figure 2B) included the same structures and connections identified in the first regional analysis, as well as connections within the superior frontal and parietal lobes. The second cluster was a left-lateralized thalamo-cortico-striatal loop, which included the inferior frontal gyrus pars orbitalis, insula, and putamen (gold in figure 2B). The third cluster (yellow-green in figure 2B) was located in the left temporal lobe. The fourth cluster included right insula and right inferior frontal gyrus (green in figure 2B). The fifth cluster was a right-lateralized occipitotemporal circuit (cyan in figure 2B). In contrast, the model examining depression while controlling for apathy yielded no edges.

**Table 6:**
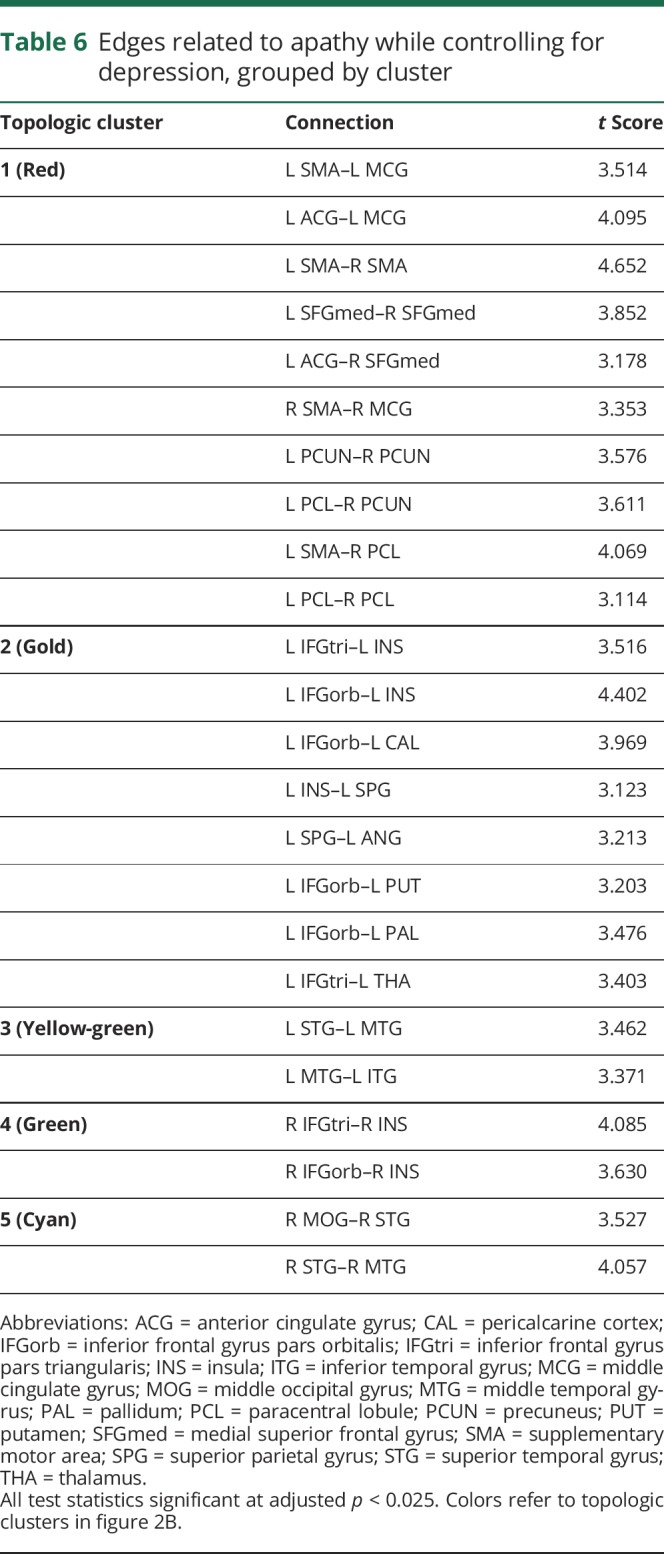
Edges related to apathy while controlling for depression, grouped by cluster

The final analysis examined edgewise correlations with apathy while controlling for depression, cognition, and education. This revealed a single cluster in the bilateral SMA, *t* = 3.396; right SMA and left superior frontal gyrus, *t* = 3.135; and left superior frontal gyrus to right precentral gyrus, *t* = 3.179 (figure 2C).

## Discussion

Our study used network analysis to examine the relationship between white matter networks and apathy in patients with SVD. We found that network measures mediated the relationship between SVD markers and apathy (figure 1), that patients with apathy, but not depression, had reduced whole-brain measures of network density and efficiency (table 5), and that this disruption could be localized to specific structural subnetworks, which included parietal-premotor, frontostriatal, and occipitotemporal connections (figure 2).

We demonstrated that apathy was not related to WMH and LIs after controlling for whole-brain network measures. Conversely, WMH and LIs only partially mediated the relationship between apathy and network measures. This novel result suggests that the parenchymal changes associated with radiologic markers of SVD, which include demyelination, gliosis, and axonal loss,^32^ may lead to apathy through the disconnection of white matter networks. This finding contextualizes previous research showing associations between apathy and radiologic markers of SVD^33,34^ and provides a plausible underlying mechanism for the pathogenesis of apathy.

Whole-brain analyses revealed that patients with apathy had lower network measures compared to the depression only, comorbid, and control SVD groups. Whole-brain networks of the apathy group were sparser and less integrated on both a global and a local scale. The density of a network reflects the overall connectivity of structures within the brain, suggesting that white matter microstructural change associated with apathy may be driven by disconnection of white matter tracts connecting regions important for motivation. Efficiency measures, on the other hand, express patterns of connectivity. A reduction in global efficiency reflects a loss of long-range connections that facilitate communication between segregated brain structures, while a reduction in local efficiency reflects a loss of short-range connections between neighboring structures.^35^

We also found that the depression group was not different with respect to the control group in terms of connectivity, consistent with findings in a different cohort of patients with more severe SVD.^2^ This does not imply that depression is not a symptom of SVD; indeed, depression was nearly twice as prevalent as apathy in our sample. It also does not imply that depression is not associated with any underlying neurobiological change. Our results suggest that the relationship between white matter microstructure and depressive symptomatology previously described in SVD^36^ may be attributable to apathy, in part because measures used to assess depressive symptomatology in these studies included apathy-related items.^2^ However, this does not preclude depression from being associated with other measures of neurobiology such as volumetric gray matter reduction or aberrant functional connectivity.

An unexpected finding of our study was that the comorbid apathy and depression group did not differ from the control group with respect to network measures; the comorbid group might be expected to show impairment similar to or worse than that of the apathy group. Furthermore, the comorbid group had higher mean scores on the AES and CESD compared to the apathy and depression only groups. One explanation for this counterintuitive effect is that the etiology of apathy differs between the apathy only and comorbid groups. For instance, apathy in our apathy only group may have been the product of vascular pathology disrupting structural networks underlying goal-directed behavior. Apathy in our comorbid group, however, could be a product of depressive symptoms,^1^ which may not result in structural network change. Thus, despite a similar behavioral presentation, the neurobiological differences between these patients with apathy may reflect different etiologies, which may have important implications for differential diagnosis and treatment. That said, the regional between-group comparison between the apathy and comorbid group yielded no significant result, possibly due to the low sample sizes in each group. Interpretations must therefore be made cautiously before replication in future studies.

We identified distinct structural subnetworks associated with apathy by examining network matrices at the level of individual edges. The bilateral SMA emerged across all our analyses, suggesting a crucial role for SMA connectivity in motivated behavior. These findings notably converge with studies of apathy in healthy individuals that have shown that higher levels of apathy were associated with reduced functional recruitment of SMA and cingulate gyrus with increasing effort levels during task-based fMRI.^37^ Furthermore, blood oxygen level–dependent signal fluctuation in the SMA during effortful trials correlated with activity in anterior cingulate gyrus, MCG, primary motor cortex, and superior parietal and frontal lobes, implying functional connectivity between these structures. This is supported by findings that apathy, but not depression, predicts the amplitude of resting-state fluctuations in the SMA of patients with Parkinson disease.^38^

The premotor cluster we found was connected to a topologic cluster that included elements of the parietal lobes, including somatosensory cortex. These were not connected with nodes in primary motor regions. These findings suggest that the parietal-premotor network, hypothesized to underlie movement intention and awareness independently of motor execution,^39^ may play a role in apathy. Electrophysiologic research in humans has shown that neural populations in premotor regions, particularly the SMA, can accurately predict a decision to move hundreds of milliseconds before the decision reaches conscious awareness.^40^ On the basis of these results, it has been proposed that internally generated behavior occurs only once neural activity in medial frontal regions reaches a certain threshold.^40^ It has been suggested that these regions transmit effort-related information directly to parietal regions, especially the somatosensory cortex.^41^ The findings of the current study suggest that functional interactions between premotor and parietal regions are supported by a structural network of white matter fibers. Pathology that damages these fibers such as SVD may result in the impaired activation or transmission of internally generated neural signals in premotor regions that precede decision-making.^40^ This may cause a failure of neural ensembles to reach threshold, leading to a reduction in goal-directed behavior that manifests clinically as apathy.

We also identified clusters in both hemispheres that included the inferior frontal gyrus pars orbitalis, inferior frontal gyrus pars triangularis, and insula. In the left hemisphere, this cluster also included the thalamus and parts of the basal ganglia, including the palladium and putamen. The putamen, as delineated by the AAL, includes portions of the nucleus accumbens. These structures and their topologic organization have been implicated in various processes that support effort-based decision-making.^42^

Reward insensitivity has been suggested to be a factor underlying apathy in stroke and Parkinson disease,^43,44^ and it is possible that this generalizes to SVD. If this is the case, then the connections in the frontostriatal network that we identified may reflect denervation of dopaminergic projections that connect the ventral striatum to the frontal lobes. Dopamine enhances willingness to exert effort for rewards,^45^ and apathy has been related to nucleus accumbens atrophy.^46^ Our results suggest that apathy may, in part, be driven by disruption of functional circuits underlying reward processing, leading to the inaccurate perception or valuation of stimuli important for decision-making.

We also found bilateral topologic clusters in the temporal lobes, with the left cluster extending to the occipital lobe. This likely reflects microstructural damage to the inferior fronto-occipital fasciculus, which has been shown in previous work.^2^ The implication of this finding is difficult to interpret because the anatomy and function of this tract remain poorly understood.^47^ Future studies are needed to investigate the relationship between the inferior fronto-occipital fasciculus and apathy.

Cognitive function played an important role in our regional findings, explaining frontostriatal and occipitotemporal, but not premotor, networks. This suggests that motivational deficits in SVD may, in part, be driven by cognitive impairment. This is consistent with cognitive apathy, a hypothesized apathy subtype characterized by impaired planning and organization, resulting in subsequent reductions in goal-directed behavior.^7^ Given the effects of SVD on cognition,^6^ it is reasonable to believe that apathy may be driven largely by cognitive deficits in this patient group. These effects, although large, do not completely explain the observed neurobiological findings with regard to SMA network connectivity. The anatomy of this network is consistent with behavioral apathy,^7^ suggesting that more fundamental deficits in behavioral activation may be present in apathetic patients with SVD.

Our findings have important clinical implications. First, they suggest that symptoms of apathy and depression, which can be challenging to distinguish clinically, can be differentiated on a neural level in SVD, implicating diffusion tensor imaging measures as a useful instrument for differential diagnosis. Second, the finding that apathy was associated with impaired cognitive function, in the context of longitudinal studies,^3^ may indicate that apathy is a prognostic factor prodromal to dementia. This highlights the importance of clinically assessing apathy; it has both immediate and long-term consequences for patients.

It must be stressed that these conclusions should be interpreted in the context of SVD before replication in other neurologic disorders. For instance, the pathophysiology of apathy might differ in neurodegenerative conditions with primary gray matter loss, in which white matter tract disconnection is a secondary concern.

A limitation of our study was in our measurement of depression, which was assessed with the CESD, a self-report instrument. Although the CESD has good reliability and validity in geriatric stroke patients,^15^ psychometric research suggests that structured clinical interviews remain the gold standard for identifying depression.^48^ Future work could more accurately diagnose patients as having major or minor depression on the basis of diagnostic criteria such as those in the DSM to examine whether these results change. This was not feasible due to our large sample and extensive protocol, although future studies can address this.

Another limitation regards our use of diffusion imaging to infer the structure of white matter pathways. Although diffusion tractography is able to reconstruct streamlines that correspond with genuine macroscopic white matter tracts,^49^ existing algorithms make it difficult to determine the precise effect of pathology on these fibers (e.g., whether disconnection is driven by demyelination, changes in membrane permeability). Despite this, tractography yields highly reproducible white matter networks in patients with SVD ^50^ that accurately predict longitudinal outcomes,^28,29^ validating its use as a clinically meaningful measure.

Our study has shown that network disruption underlies the relationship between radiologic markers of SVD and apathy. In addition, patients with SVD with apathy have white matter networks that are sparser and less efficient compared to those in other patients with SVD. We also demonstrated a clear dissociation between the underlying neurobiology of apathy and depression in SVD; patients with depression showed no impairment on whole-brain measures of network integrity compared to controls with subthreshold levels of neuropsychiatric symptomatology. We also localized apathy to parietal-premotor and frontostriatal networks. The anatomy of these networks is consistent with functional studies that show parietal-premotor regions to be associated with volitional behavior and frontostriatal regions to be associated with reward processing. Future studies could investigate the topology of these specific networks a priori with focused experimental hypotheses and how these change longitudinally. The connectivity of these white matter networks also offers a potential biomarker for detecting motivational deficits in neurologic disorders.

## Glossary

AAL: Automated Anatomical Labeling
AES: Apathy Evaluation Scale
ANCOVA: analysis of covariance
CESD: Centre for Epidemiological Studies Depression Scale
DSM-IV-TR: *Diagnostic and Statistical Manual of Mental Disorders, 4th edition, text revision*
EPI: echo-planar imaging
FLAIR: fluid-attenuated inversion recovery
LI: lacunar infarct
MCG: middle cingulate gyrus
NBS: Network Based Statistic
RUN DMC: Radboud University Nijmegen Diffusion Tensor and Magnetic Resonance Cohort
SMA: supplementary motor area
SVD: small vessel disease
TE: echo time
TI: inversion time
TR: repetition time
WMH: white matter hyperintensities
